# An Information App (e-TOP) to Support Parents’ Information Needs During the First Year at Home After Preterm Birth: Development and Usability Study

**DOI:** 10.2196/75569

**Published:** 2025-10-22

**Authors:** Monique Flierman, Eline L Möller, Vincent L Vijn, Raoul H Engelbert, Martine Jeukens-Visser, Anton H van Kaam, Daniel Bossen

**Affiliations:** 1 Department of Rehabilitation, Amsterdam Reproduction and Development, Amsterdam UMC Location University of Amsterdam, Meibergdreef 9, Amsterdam, 1105 AZ, The Netherlands, 31 622947431; 2 Centre of Expertise Urban Vitality, Faculty of Health, Sports and Physical Activity, Amsterdam University of Applied Sciences, Amsterdam, The Netherlands; 3 Department of Rehabilitation, Amsterdam University Medical Centers, Amsterdam, The Netherlands; 4 Faculty of Digital Media and Creative Industry, Amsterdam University of Applied Sciences, Amsterdam, The Netherlands; 5 Amsterdam University Medical Center, Emma Kinderziekenhuis, Amsterdam, The Netherlands; 6 Department of Neonatology, Amsterdam Reproduction and Development, Amsterdam UMC Location University of Amsterdam, Amsterdam, The Netherlands

**Keywords:** mHealth, premature infants, postdischarge care, usability, user experience, parental support, app, mobile health

## Abstract

**Background:**

Parents of preterm infants often face challenges in transitioning from hospital to home, requiring reliable and accessible information to support their caregiving. Mobile health interventions have the potential to supplement postdischarge education and empower parents by providing tailored, evidence-based information.

**Objective:**

The primary objectives of this study were to develop an information app (e-TOP) using a participatory design approach, incorporating input from parents and health care professionals, and evaluate its usability.

**Methods:**

A 2-phase, mixed methods design was used for this study. In Phase 1, the app was developed through iterative focus group discussions with parents of preterm infants, parents with limited health literacy, and TOP interventionists. In Phase 2, a usability study was conducted over 6 months with parents of preterm infants. Usability was assessed with a broad range of measurements: thinking aloud sessions, user engagement analytics, 2 questionnaires, the system usability scale (SUS), a customized satisfaction survey, open question sections, and semistructured interviews.

**Results:**

The collaborative approach with end users and experts for the development led to a fully functional e-TOP app. Expert review and content validation ensured that information was clinically accurate, accessible, and relevant to parental needs. For the usability testing, a total of 58 families (116 participants) were recruited and 69 participants actively used the app. The median cumulative e-TOP usage per participant in 6 months (26 weeks) was 39 minutes (IQR 8.8‐53.0). The median number of actions was 64.0 (IQR 33.5‐88.0). The e-TOP app received a median SUS score of 75 (IQR 67.5‐80.0), indicating good usability. Participants rated their overall median satisfaction at 7.0 (IQR 7.0‐8.0) out of 10. While the app was perceived as useful for finding information on prematurity-specific topics, engagement declined over time. Qualitative feedback highlighted a need for improved navigation (eg, a search function), expanded content (eg, motor development and sensory processing), and more interactive features (eg, chat support and parental community forums).

**Conclusions:**

The e-TOP app is a valuable digital resource that can supplement postdischarge care by providing tailored, evidence-based, and accessible information for parents of preterm infants. While usability scores were high, engagement trends and retrieved feedback suggest the need for enhanced retention strategies, such as push notifications, timeline-based navigation structure, interactive tools, and adding (practical) content. Future iterations to improve the inclusivity of the e-TOP app require strategies to engage fathers and parents with limited health literacy skills.

## Introduction

### Background

In the Netherlands, 6.9 % of the children are born prematurely (before 37 weeks of gestational age) [1]. Although advances in perinatal and neonatal care have significantly improved survival rates for preterm infants [2], preterm birth still accounts for 66% of all deaths occurring before or during the first month after birth [3]. Prematurity also has large adverse effects on children’s motor, cognitive, and socioemotional development [4]. Giving birth to a premature infant is also a disruptive start for parents [5]. Parents of preterm infants often experience a range of mental health problems, such as anxiety and depression [6]. The dependency on medical and health professionals can challenge the formation of a secure infant-parent relationship and parents’ self-efficacy in their ability to independently care for their infant [7]. The transition to home following discharge is known as challenging. Without the 24/7 access to health care professionals, parents of preterm infants feel unprepared for this new situation and lack confidence in their capacity to care for their infant [8]. Despite efforts to improve parents’ readiness for discharge and the availability of follow-up services, many parents of preterm infants continue to experience unmet needs for support and practical information after leaving the hospital [9,10]. As the infant’s care needs and developmental milestones evolve during the first year, the informational needs of parents of preterm infants also shift accordingly. Providing tailored information about the consequences of prematurity and the specific health, behavioral, and developmental needs of premature infants can strengthen parental self-efficacy, enhance their sense of security in caring for their infant, and potentially reduce health care use [11,12].

### Search for Information

After returning home, parents of preterm infants search the internet for information on topics, such as infant crying, choice of formula, common illnesses, and developmental stages. While they use search engines, social media, or mobile health (mHealth) apps, finding appropriate and reliable information on the unique challenges of caring for a preterm infant at home remains difficult [8,9]. The use of mHealth could support a shift from time- and professional-dependent access to information toward a more autonomous approach, empowering parents to obtain information whenever and wherever they need it. However, for mHealth apps to be useful and effective, they must address the specific information needs of parents of preterm infants [13,14]. In addition, the app must be user-friendly [15] and the information accessible and easy to understand for parents across varying levels of education and health literacy [16].

### mHealth Apps

mHealth education interventions for parents of premature infants were developed over the last decades. However, Richardson’s evaluation of mobile apps targeting parents of infants in the neonatal intensive care unit (NICU) revealed that many apps are lacking quality and credibility or do not contain content identified by parents as important [17]. In contrast, the study on the NICU2HOME app provides high-quality information on infant and self-care during and after the NICU stay and showed promising results in enhancing parental self-efficacy and discharge preparedness [18]. Despite the rich body of literature regarding parental needs for information after discharge, available apps targeting the postdischarge period are limited. A systematic app review of mobile information apps for parents of preterm infants after hospital discharge found that, while the overall quality of the identified apps was acceptable, they insufficiently addressed key postdischarge concerns, such as information on transitions in nutrition, sleep patterns, or developmental stages [19]. This lack of targeted content may contribute to the limited usage and adoption of these apps, a common pitfall when end users and stakeholders are not adequately involved in the design process [20]. Participatory design approaches, such as the design thinking method, ensure that user requirements are accurately captured from the perspective of the end users, thereby enhancing usability and facilitating effective implementation [21-24].

### The Dutch Context: TOP Program

In the Netherlands, a 1-year home-based responsive parenting intervention (TOP program) is standard care for very preterm infants and their parents and is currently expanded for moderate to late preterm infants. Pediatric physical therapists with an additional 1-year TOP training and affiliated with the Dutch Expertise Center for Premature Infants (EOP) carry out the TOP program. The strength-based and process-oriented intervention consists of 7 key strategies to enhance parental understanding of the infants’ behavioral and developmental needs [25]. An important key strategy is to provide parents with information about their infant’s behavioral and developmental needs. To enhance parents’ knowledge and understanding of their infants’ needs, TOP interventionists provide verbal information transfer and an individualized written parental report with strength-based recommendations accompanied by photos taken during the home visit. Despite the benefits of face-to-face care, there is a need for a complementary mHealth intervention that enhances information retention, supports parental confidence in managing their infant’s care, and potentially reduces their reliance on health care professionals [9,14]. Therefore, we aim to complement and enhance the existing Dutch TOP program by addressing gaps in information retention and supporting professionals in delivering reliable information to parents. Before conducting more extensive research to measure its effectiveness, developing an information app that ensures that user requirements are effectively incorporated and is tailored to the specific needs of parents of preterm infants is crucial.

The aim of this study was two-fold: (1) to develop a postdischarge information app (e-TOP) for parents of premature infants and (2) to evaluate its usability.

## Phase 1: Development of the e-TOP App

### Phase 1 Methods

#### Setting

The study was performed between October 2021 and November 2023 by the EOP, affiliated with the Amsterdam University Medical Center, in collaboration with the Amsterdam University of Applied Sciences.

#### Ethical Considerations

The study was approved by the Medical Ethical Committee of the Stichting Amsterdam UMC (NL78996.018.21). All participants received an information letter detailing the study’s objectives, timeline, content, target group, data management practices, and privacy considerations. Subsequently, informed consent forms were obtained. Participants were officially enrolled in the study upon providing written consent. Data were pseudonymized. Participants did not receive any compensation.

#### Participants

The user-centered design approach for the development of the prototype started with 3 focus groups, 1 with TOP interventionists and 2 with parents of preterm infants. A pragmatic sampling approach was used, drawing on available data from the EOP center to identify and invite participants (TOP interventionists) with relevant experience across different contexts [26]. For focus group 1, TOP interventionists with more than 5 years of experience, representing different geographic and socioeconomic regions, were approached. For the parent focus groups, participating TOP interventionists approached potential eligible and interested families within their caseload. Parents were included if they (1) participated in the TOP program for at least 3 months and (2) were able to understand and speak the Dutch language. Focus group 3 specifically involved parents of preterm infants with limited health literacy (LHL). These parents were identified and recruited by the TOP interventionist by using the following 2 additional criteria: (1) parents struggle with understanding and applying the information provided during the intervention or in the parent report and (2) fulfill the criteria for LHL according to the Dutch checklist to recognize LHL [27].

#### Focus Group Meetings

The script for the focus group meetings was developed collaboratively with the research team, guided by the 2 main objectives of the sessions (Multimedia Appendix 1). First, to verify the identified need for information on the 10 topics previously found in our qualitative research [13] and to enrich these with participants’ personal experiences. Second, to elicit preferences regarding content, topics, design, and navigation of the app.

To address the first objective, the research team formulated open-ended questions and prompts for each of the 10 topics. These were designed to stimulate reflection, encourage sharing of personal experiences, and allow for the addition of new themes. Examples and short scenarios were incorporated to help participants relate the topics to their own context.

**Figure 1.**
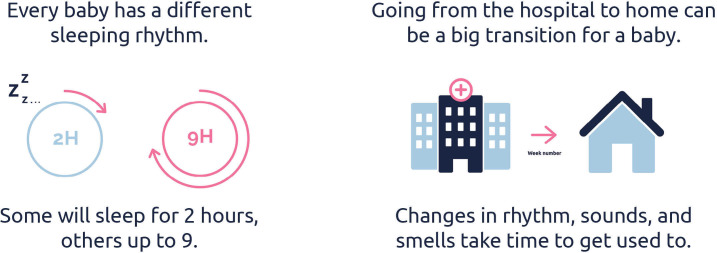
Information cards.

**Figure 2.**
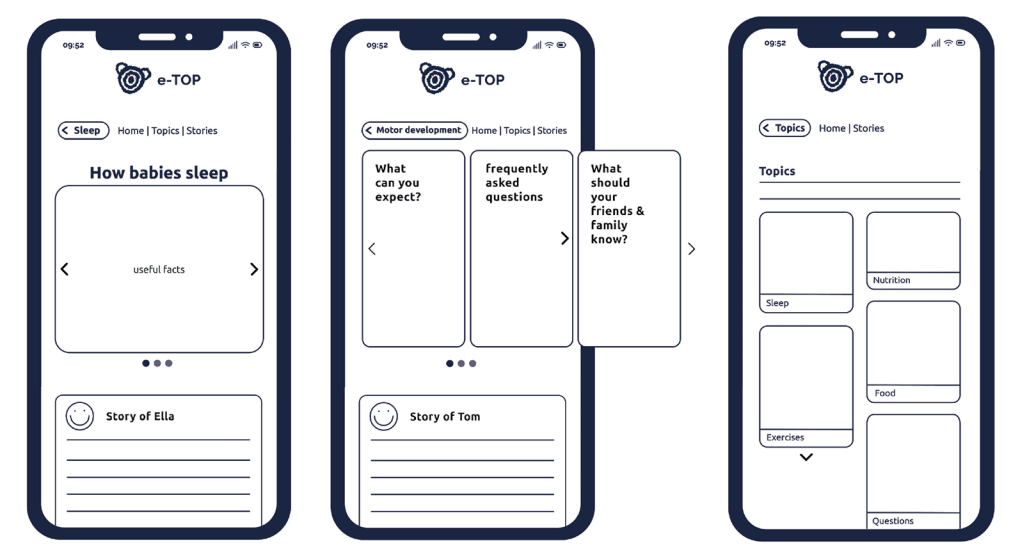
Mock-up.

For the second objective, the script included guided activities involving visual and textual materials, such as screenshots of existing parenting apps, information cards, mock-ups, and text samples. These were selected and designed by the research team to support discussion on specific design features (eg, tone of voice, level of detail, and visual presentation) and to facilitate comparison between different styles. See Figure 1; information cards and Figure 2; mock-ups.

The draft script was reviewed in 2 rounds by the research team to ensure alignment with the study objectives, clarity of language, and feasibility within the planned 2-hour sessions. Minor adjustments were made following pilot testing with a small group of colleagues to refine the sequence of questions and ensure smooth transitions between topics.

We conducted 3 focus group sessions. In the first meeting, 8 female TOP interventionists participated. Five interventionists worked in an urban setting with a diverse population in a challenging socioeconomic environment.

In the second focus group, 9 parents (5 mothers and 4 fathers) participated. Their mean age was 35.6 years (SD 5.8). For 5 parents, it was their firstborn, and for the other 4 parents, it was their second child. Their infants’ mean gestational age was 30 weeks and 2 days (SD 19.0). In the third focus group meeting, 6 mothers with LHL participated with a mean age of 31.6 years (SD 2.2). For 5 mothers, it was their firstborn infant, and for 1 mother, her third child. Their infants’ mean gestational age was 29 weeks and 1 day (SD 21.8).

The three 2-hour online focus group meetings were organized in the evenings, which was indicated by participants as the most convenient time. The focus groups were facilitated by an independent female researcher, MM. She was not involved in the study design or analysis. The first author (MF) attended the focus groups to take field notes but did not lead the discussions. The 3 focus group meetings were audio-recorded, relistened to, and summarized. Findings were discussed within the research team.

### Phase 1 Results

#### Overview

The results of the focus group discussions with TOP interventionists and parents provided valuable insights that shaped the development of the e-TOP app, directly influencing its content and design refinements. TOP interventionists recommended not to use the age-based developmental norms strictly**,** as individual variation among preterm infants is substantial. Instead of providing medical guidance, the app should direct parents to contact their general practitioner or pediatrician when necessary. Additional topics identified by participants were the digestive system, reflux, constipation, medication management, sensory overload and stimulation, parent-infant attachment, and bonding. Parents expressed a preference for clear, supportive, and actionable information. Information should have a layered content structure, allowing simplified messages and scientifically substantiated information. A summary of these findings is provided in Table 1. We used them as a guideline for the further development of the e-TOP app.

**Table 1.** Results of focus group meetings.

Topics	Focus group 1^a^	Focus group 2^b^	Focus group 3^c^
Participants’ associations with a digital information source.	Reliable and user-friendly digital source aligns well with the needs of young parents.	An app can offer practical guidance to support everyday care. Given the limited experience of general practitioners and Dutch health services with prematurity, the app could also be a valuable source.	Participants expressed curiosity and recognized the usefulness of an app in addressing their concerns.
What questions do parents have?	Ten topics most frequently searched align with participants’ concerns, validating the app’s content. In addition, practical information on adequate sensory input and the use of slings is needed.	Different experiences with gavage feeding at home, a need for more tailored information. What can I do with my premature infant?	Participants sought information on baby positioning, especially with the preferred head position. In addition to the shown topics: reflux and gavage feeding tips. Information about constipation.
About which topics do parents seek additional information?	Information about constipation, intestinal cramps, reflux, or medication.	Feeding, sleep patterns, sensory overexcitability	Inconsolable crying. Psychological support for anxiety. Premature clothes. Information about long-term effects of oxygen.
What support do parents need?	Finding psychological support for themselves.	Need for a readily accessible professional after discharge.	Information on available services, particularly for single moms.
Where do they look for information?	Communities including Facebook groups and consumer association websites.	Parents shared their favorite websites, but they are often not specific to premature infants.	Forums such as 24baby are used. Other parents with experiences serve as key sources of information.
Presentation of information	Adaptability to literacy levels. Videos and animations in multiple languages for less literate parents.	Varied content preferences. Different preferences: some participants liked text-based information, others favored videos.	Preference for animation (visually oriented). Practical demonstrations with videos for making the crib. Interactive features such as a chat box for quick response. Peer experiences. Some valued real-life success stories; others were not interested in fellow sufferers.
Navigation	A structured topic design, with an FAQ chapter.	Search button to improve content discoverability.	Clicking through for more detailed information.
Information cards	Reassuring and supportive tone is essential. Normalizing and comforting tone of voice is important.	Concise messages can raise questions. The messages should not oversimplify.	Informative, necessity to be able to search further for more extensive information.
Text validation	Be aware of the tone of voice, clarity of the information.	Comforting information is important. An informal tone of voice is nice, it relates to parents and feels personal.	Easy-to-read text, supplemented by practical examples on what to do. Information can be more casually read, like you talk to a trusted friend or relative. It needs to be valid information.
Depth of information	Layered information structure. Parents need to be able to explore in-depth details or stay at a simpler level.	Different headings can help to make content more accessible.	Information should clearly explain the differences in care for premature infants.
Scope of information	Coverage for the first year, aligning with the TOP program.	Guidance on more complex cases should refer parents to the health care professionals. Potential integration with a hospital app could enhance usability.	Developmental milestones per week. Receiving updates about what your baby is doing in relation to other infants (corrected for prematurity)

a TOP interventionist.

b Parents of preterm infants.

c Parents of preterm infants with limited health literacy.

#### From Prototype to a Functional e-TOP App

To create content for the app, experts were recruited through the researchers’ network. Health care experts were selected based on their expertise regarding prematurity or related topics, such as feeding, sleep, or motor development. To address user needs, the following 4 steps were taken. First, the topics parents indicated in our prior research [13] were complemented with the findings of the focus group meetings. Experts drafted comprehensive information on these topics. Two members of the research team (MF and EM) finalized the content in 3 rounds of review and refinement. Second, the Dutch Expertise Center for reducing health inequities provided support to improve the readability, whereafter the text was simplified by the first 2 authors (MF and EM) to that of the reading ability to understand everyday health information (B1/B2). Key messages for each topic were formulated at level A2 for parents who prefer to read short sentences with familiar words[28]. In addition to text, a read-out-loud function was added. Third, the first 2 authors (MF and EM) included images and picture stories. Fourth, video content, either developed by health care experts from different fields or retrieved from the EOP library, was added. Videos consisted of interviews, demonstrations of parent-infant interaction, and explanations of infant behavioral signs. To clarify the content, videos had voice-overs and subtitles.

**Figure 3.**
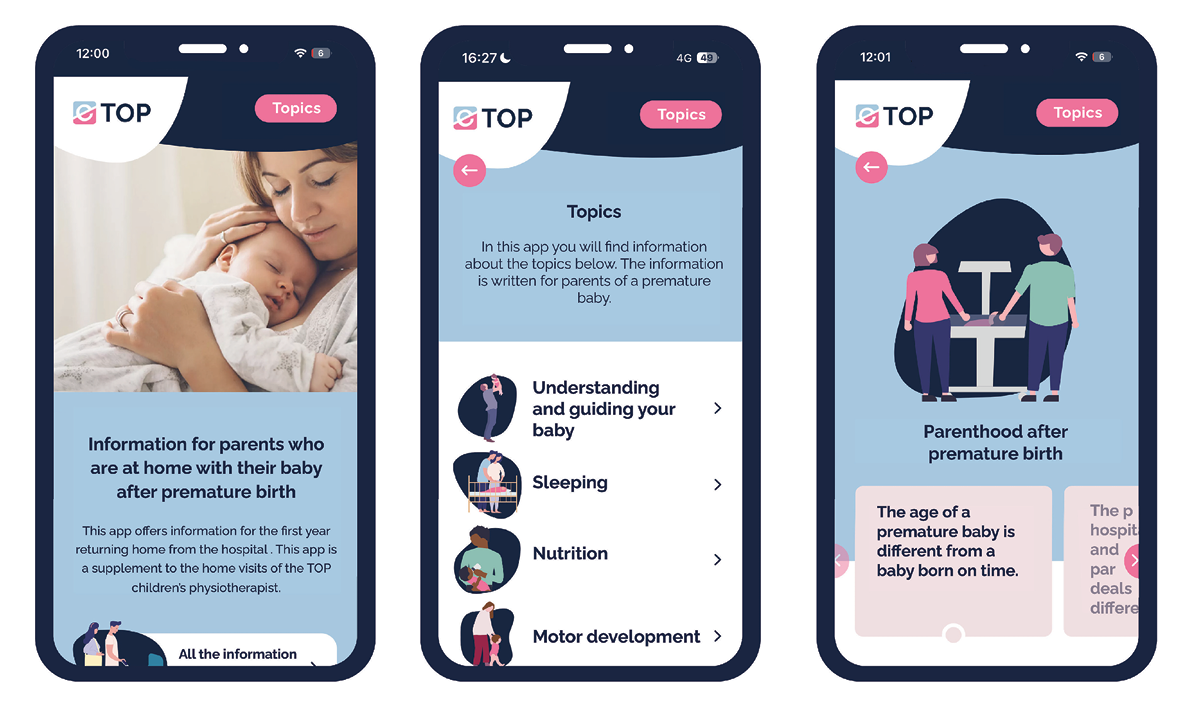
e-TOP app.

Simultaneously with creating content, we collaborated with a design company (ZIGT) to build a functional prototype of a web app accessible on smartphones, tablets, and desktop devices. The research team organized and posted all content leading to the final functional e-TOP app (see Figure 3).

## Phase 2: Evaluation

### Phase 2 Methods

#### Participants

For the evaluation of the e-TOP app, the sample was composed of parents with a very preterm born infant (below 32^0/7^ gestation) and moderate preterm born infants (32^0/7^-34^6/7^ weeks of gestation). Parents of very preterm infants received the TOP program (routine care, reimbursed by the basic package of insurers), and parents of moderate preterm infants received a shortened TOP program in a study context [29]. In addition to the home visits by the TOP interventionists, all participants received access to the e-TOP app.

#### Procedures

TOP interventionists were recruited using an invitation email distributed by the EOP center. Fifteen TOP interventionists responded to be available for participation in the study. They received a 1-day training in the intervention and research protocol including the informed consent procedure. The enrollment took place between November 2022 and March 2023. TOP interventionists introduced the study to eligible parents within their caseload. The requirement for parents to take part in the study was to be able to read and speak Dutch. When interested, parents received an information letter from the study center. If they agreed to participate, an informed consent visit was scheduled. Upon signing the informed consent, both parents received a unique access code and an animation video explaining how to install the e-TOP app. In addition, at the start (T0), parents were invited via email to complete a questionnaire on sociodemographic information including age, educational level, employment status, and family structure. Parents had continuous access to the app for the duration of 6 months. During this period, Matomo analytics was used to collect user data and thinking aloud tests were performed. At the end of this period (T1), parents received a follow-up invitation to complete questionnaires assessing their experiences, and 10 participants were invited for an interview.

#### Usability

For a comprehensive evaluation of the usability of the app, we used qualitative and quantitative measurements. User data provided insights into objective usage engagement, while qualitative data highlighted usability needs and identified issues for further improvement [30].

##### Usability Testing: Thinking Aloud

Thinking aloud tests were used to gain insights into the ease of use and navigation experiences with the app [31,32]. While navigating the app, parents were encouraged to express their thoughts, frustrations, questions, and feedback in real time. The tests were conducted by 2 students from the faculty of health of the University of Applied Sciences Amsterdam, supervised by author VV.

For the sample technique, a stepwise approach was used to ensure that the final participant list for the thinking aloud session included a varied sample across different levels of app engagement. The selection process involved the following steps: (1) all families were initially screened for e-TOP app installation. (2) For each participant, the total app usage time (in seconds) was calculated. (3) Quartiles of app usage time were determined, and participants were divided into 4 usage groups. (4) Each group was randomly ordered using a randomization tool. (5) Three participants per group (n=12) were approached by email for participation.

Participants were asked to complete 3 tasks: (1) determine when your infant can pick up a small object, (2) identify what actions you can take as a parent when your child is anxious about touch, and (3) calculate your infant’s corrected age. After completing each task, participants rated the difficulty using the Single Ease Question [33], which scores task performance on a scale from 1 (very difficult) to 7 (very easy). Afterward, parents were asked questions regarding the content (eg, “Are there any topics you would like to add?”), usability (eg, “How was your experience with the e-TOP app?”), and comprehension (eg, “Did you understand the answers you found when searching for information?”). These sessions were screen-recorded, and audio transcripts were generated.

##### User Data

User data were monitored and analyzed with the web analytics tool Matomo [34]. Users were defined as those who accessed the e-TOP app more than once. The main metrics of interest were installs, visits, visit duration and change of usage over time, and visited topics.

To allow participants to provide immediate feedback on the app, the e-TOP app included a prompt at the end of each page asking, “Was this information helpful?” with 2 response options: yes or no. After selecting an option, a comment box appeared, allowing users to provide additional feedback.

##### Usability Questionnaires

The Dutch version of the system usability scale (SUS) was used [35]. The SUS is an easy-to-use, valid, and reliable questionnaire for usability assessment of mHealth apps. The scale consists of 10 statements, which can be completed within 10 minutes. All items are scored on a 5-point Likert scale from “strongly disagree” to “strongly agree.” Scores per item are converted to an overall SUS score, which ranges from 0 to 100, with higher scores indicating better usability. Overall scores from 0 to 50 indicate “not acceptable,” scores from 51 to 67 indicate a marginal level of usability, and scores from 68 to 100 indicate “acceptable to excellent” levels of usability [36].

To measure user experience and appropriateness of the content, a customized 10-item questionnaire was used. Seven statements were scored on a 5-point Likert scale from “strongly disagree” to “strongly agree.” Two statement examples are: “The e-TOP app helps me to find information about premature birth” and “The pictures and videos help me to understand the information.” Overall satisfaction with the app was scored on a numeric scale from 1 to 10 with 1 indicating very dissatisfied to 10 indicating extremely satisfied. Two open-response questions allowed participants to provide unrestricted and more subjective answers: “What information did you miss when using the e-TOP app?” and “Do you have suggestions to improve the e-TOP app?”

##### Interviews

The interview guide was developed in collaboration with the research team. Questions were formulated to address predefined aspects considered critical for evaluating the app’s usability. These aspects were derived from the study objectives and focused on identifying strengths, limitations, and opportunities for improvement.

To gain a more in-depth understanding of the experiences with the e-TOP app, a subgroup of participants (n=10) was invited for a semistructured interview with the first author (MF) to discuss their experiences with the e-TOP app in more detail. Purposeful sampling, including educational level, gender, and e-TOP app usage, was used to obtain a heterogeneous sample.

Interviews were scheduled at a time convenient for the parents (by phone or video call). The interview guide was set up to elicit direct, actionable feedback on app usage (Multimedia Appendix 2).

### Data Analysis

Questionnaire data were collected using the Castor database (Electronic Data Capture, Ciwit BV, 2021). Descriptive statistics were used to describe the participants and the user data (installs, visits, visit duration and change of usage over time, and visited topics). Questionnaire data were checked for normal distributions using a graphical summary of data and descriptive statistics. The median score with IQR was used in case of skewed distributions.

For the qualitative data collected through thinking aloud sessions and interviews, a pragmatic thematic analysis approach was used [37]. For the thinking aloud analysis, initial open coding was analyzed and discussed within the project group and subsequently categorized into themes. Subthemes were then developed under each main theme to capture more specific insights. In addition, user comments and open questions from the questionnaires were evaluated and categorized by the first author (MF). Ultimately, the qualitative analysis resulted in 4 overarching themes: user experience, content, navigation, and functionality.

### Phase 2 Results

#### Participants

**Figure 4.**
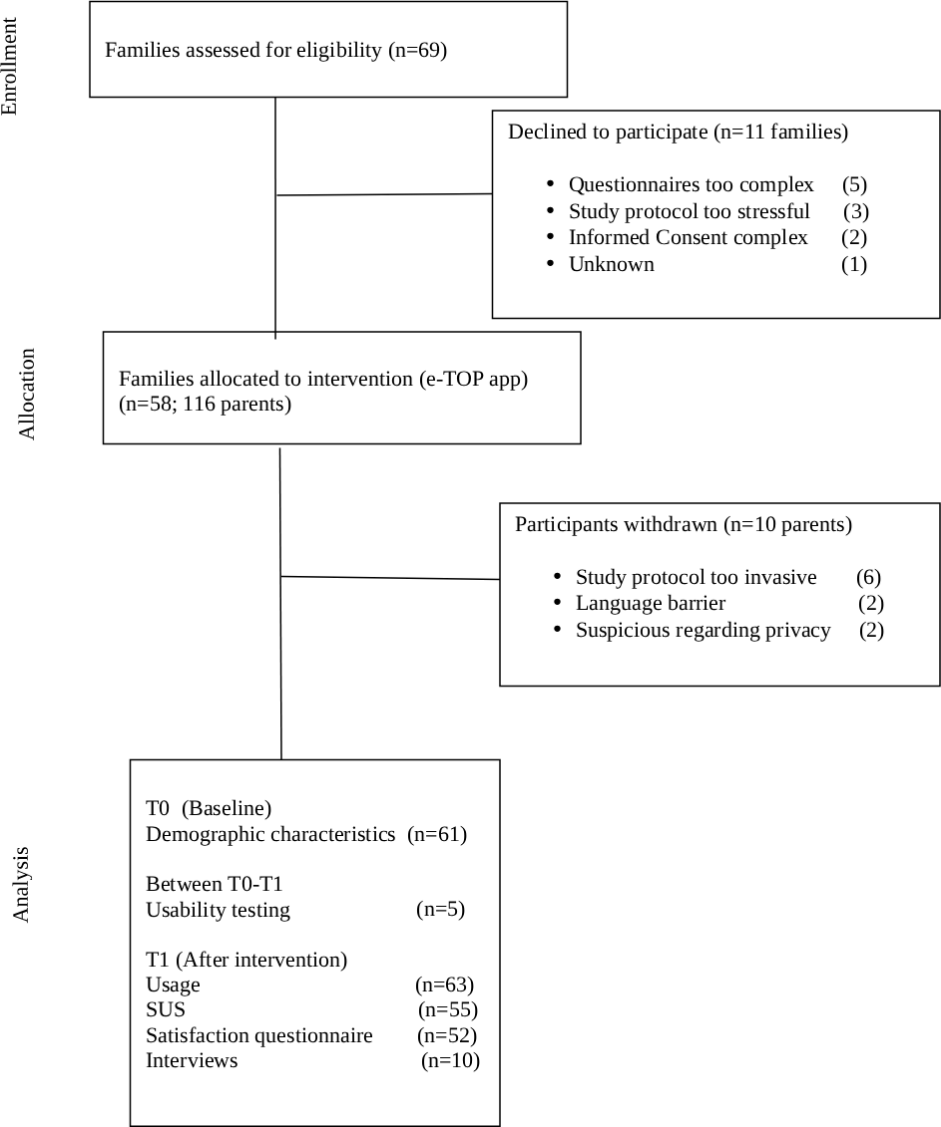
Flow of participants. SUS: system usability scale.

A total of 69 families with a preterm infant were assessed for eligibility by 15 TOP interventionists. Of these, 58 eligible families, consisting of 58 fathers and 58 mothers, agreed to participate, signed informed consent, and received the access code for the e-TOP app. Five families (10 participants) withdrew from the study after signing informed consent and receiving the first questionnaires. At T0, 61 participants completed the demographic questionnaire (see Figure 4 for flow diagram). For participant characteristics, see Table 2.

**Table 2.** Participant characteristics.

Participant characteristics	Values, n (%)
Parent	
Mother	42 (69)
Father	19 (31)
Family status	
Firstborn child	37 (61)
Family status of 2 parents	61 (100)
Language	
Dutch language for health care information	60 (98)
Education^a^	
Lower	2 (3)
Intermediate	22 (36)
High	37 (61)
Employment	
Full-time	35 (57)
Part-time	25 (41)
No employment	1 (2%)

a Highest educational level completed: lower level education refers to primary school, vocational education, lower of middle general secondary education. Intermediate: refers to higher secondary general education, preuniversity education. High: higher vocational or university.

#### Usability Results

##### Thinking Aloud

Ultimately 5 participants (3 fathers and 2 mothers) were willing to schedule an appointment to perform the Thinking aloud tasks. The ease of use was rated as follows: Task 1 had a mean score of 6.4 (SD 0.55), Task 2 a mean of 6.0 (SD 0.89), and Task 3 a mean of 6.0 (SD 1.22) on a scale from 1 to 7, indicating that all 3 tasks were easy to perform. Participants appreciated the app, the tone of voice, and the understandability of the information. Participants provided explicit and actionable recommendations regarding content and functionality. For example, regarding content, participants expressed a desire for more information on health issues and medication. They also expressed interest in instructional videos on techniques, such as holding and carrying their baby. Regarding functionality, a home button, a scroll arrow, and a search function were missed.

##### User Data

**Figure 5.**
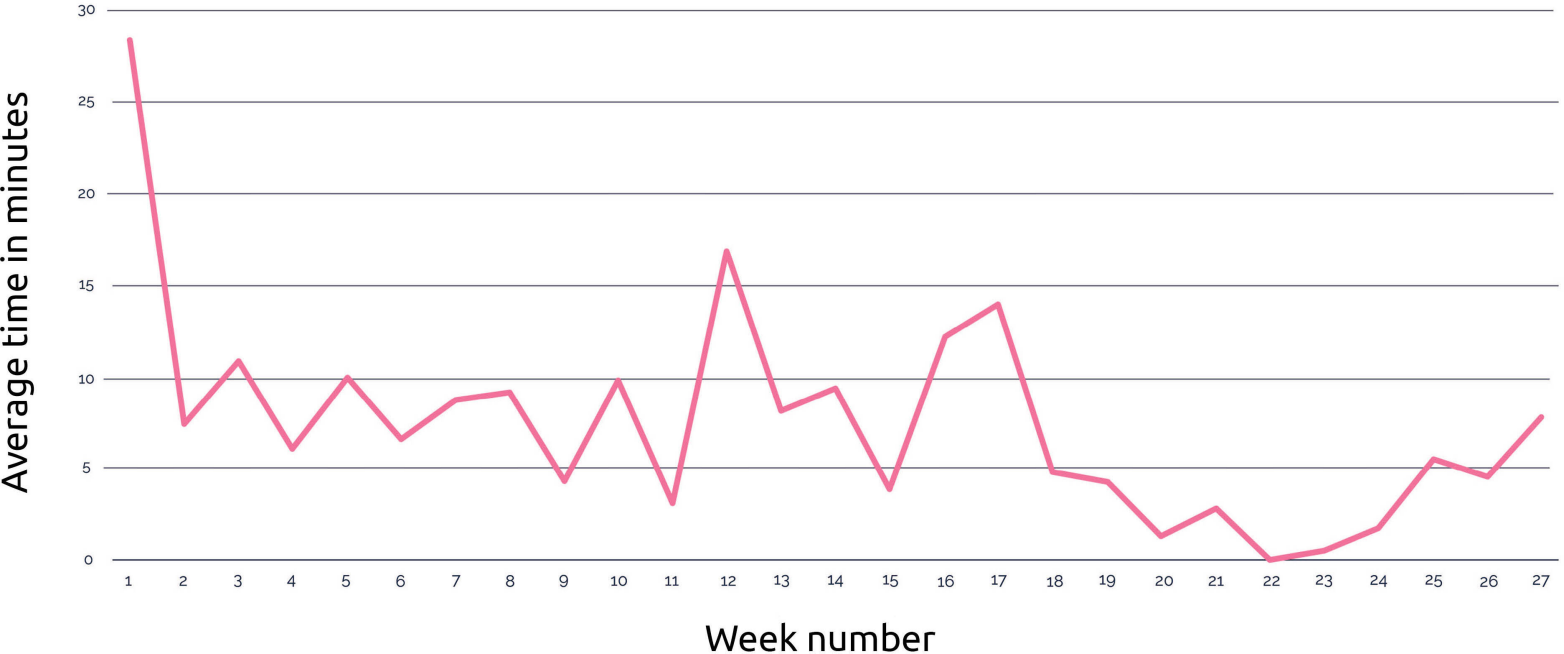
Time spent per week.

In total, 69 participants (47 mothers and 22 fathers) used the personal access code to log in. Due to ad blockers, tracking data were available for 63 participants. The median cumulative e-TOP usage per participant during 6 months (26 weeks) was 39 minutes (IQR 8.8‐53.0). The median number of actions was 64.0 (IQR 33.5‐88.0). Most actions and the largest time spent on the app were registered during the 2 weeks after receiving the access code. See Figures5  6 for further details. Multiple participants visited all 10 topics during the first 2 weeks. The total amount of visits per topic declined after this period with another peak in visits between weeks 11 and 17. The most visited topics in the app were nutrition, sleep, and correcting age for prematurity (Table 3).

**Figure 6.**
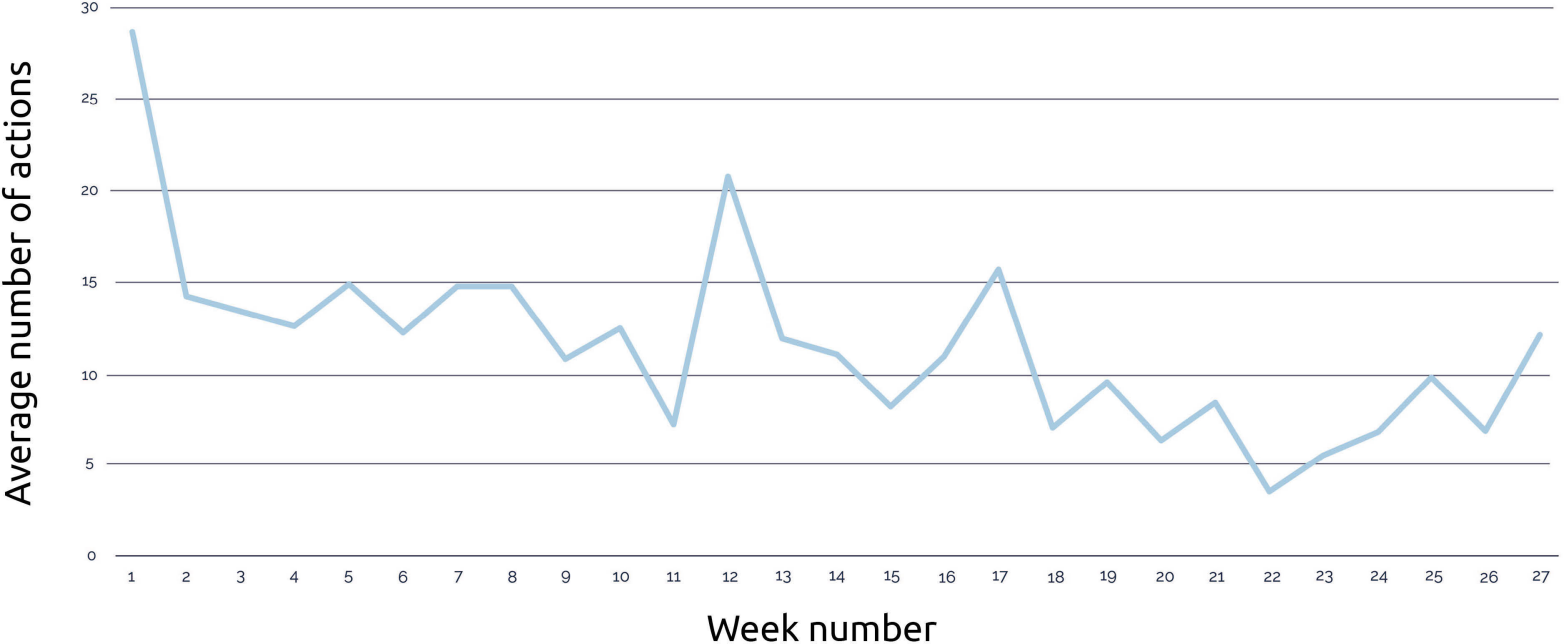
Actions per week.

**Table 3.** Visited topics on the e-TOP app.

Topic	Total number of visits	Peak visits (week)
Nutrition	81	17
Sleep	79	6
Correcting age	54	3
Understanding your baby	49	4
Long-term consequences	44	14
Motor development	42	11
Returning to work	33	4
Parenting	25	4
Infant health	23	4
Follow-up	21	9

### Usability Questionnaires

A total of 55 participants completed the SUS, yielding a median total SUS score of 75.0 (IQR 67.5‐80.0), indicating generally good usability. The app was rated positively for its ease of use, functional integration, and user confidence.

Participants gave the app an overall satisfaction of 7.0 (IQR 7.0‐8.0), on a scale of 1‐10. Content satisfaction was rated with a mean score of 3.7 (SD 0.89) and the app’s appearance was considered attractive, with a mean score of 3.6 (SD 0.83) on a scale from 1 to 5. Participants found the pictures and videos particularly helpful (mean 4.0, SD 0.56) and expressed that the app was helpful in addition to face-to-face care from the TOP interventionist (mean 3.5, SD 0.99).

### Qualitative Data

Data collected through thinking aloud sessions, interviews, and written comments from the app’s comment box were categorized in 4 themes: user experience, content, navigation, and functionality (see Table 4).

**Table 4.** Integrated results matrix for quantitative and qualitative data.

Themes	Quantitative findings (questionnaires and thinking aloud)	Qualitative findings (interviews and comment box)	Meta-inference
User experience	Overall satisfaction: median 7.0/10 (IQR 7-8) Visual attractiveness: mean 3.6/5	Users appreciated the depth and specificity of information and the app’s tone. However, they missed tailored responses and desired more interactive features.	Parents appreciate the user experience, yet desire more individualized and interactive features.
Content	Content rating: mean 3.7/5 (SD 0.89) Visual helpfulness: mean 4.0/10 (SD 0.56)	Content was viewed as highly relevant and unique to prematurity. Suggestions included more on motor development, practical care, and instructional videos.	Content meets core needs, but parents want more concrete, in-depth, and visually supported information tailored to daily care.
Navigation	Median total SUS^a^ score of 75.0 (IQR 67.5‐80.0)	Parents found the app easy to use and appreciated its tone; they missed a search button and clearer navigation	Usability is high, but navigation challenges (especially in locating specific info) call for interface redesign and better structure.
Functionality	Task ease thinking aloud: mean 6.1/7 (SD 0.89) Usage: median 39 minutes (IQR 8.8-53) over 6 months	Parents appreciated the functions in the app, but had some suggestions for improvements.	Functionality is strong but could be elevated by improved orientation, content sharing, and age-specific organization (eg, timeline).

a SUS: system usability scale.

### User Experience

Participants appreciated the specificity and depth of information provided yet expressed concerns about retrieving tailored answers to specific situations. As a participant in the interview stated, “The only thing I couldn’t find was whether it’s possible to swim with a premature baby and at what age that is safe”. The app facilitated users’ understanding of infant behavioral cues and sleep patterns. However, parents also preferred direct interaction with a TOP interventionist, indicating a desire for more personalized and human support. For example, a mother stated in the interview that “The TOP interventionist explained it to me personally, so I found that easier and more comfortable.” Suggestions were given to incorporate interactive features, such as chat support or a parent community, indicating a preference for increased interactivity and social connection within the app.

### Content

The content was generally deemed useful and relevant to the specific needs of parents with premature infants. As 1 participant noted in the comment box, “It gives information you cannot find on the internet, all content is related to premature birth.” Suggestions for improvement emerged from both the interviews and the comment box. Requests for additional practical tips, along with more in-depth information on motor development and health-related concerns, highlighted opportunities to expand the scope of information. As a participant stated during the interview: “More information about motor development, like crawling, creeping, and sitting, would be helpful.*”* Another parent expressed during the interview, "I would like there to be more concrete information, such as a chart with a baby weighing this much should drink this much.” Videos were highlighted as particularly effective for conveying complex concepts, such as sleep patterns and infant signals.

### Navigation

While users found the landing pages clear and well-organized, they reported difficulties with lengthy chapters and locating specific information. As a participant during the interview reported: “Searching for specific information is more difficult if you don’t know under which chapter to search.” Another participant reported in the comment box: “The sub-chapters contain too much information and not so easy to find answers to your questions.” The need for enhanced navigation design, such as the inclusion of a search function, a home button, and scroll arrows, was suggested and could enhance information retrieval. A participant explained during the thinking aloud session: "If there was an arrow next to the app indicating that there’s more room to scroll down, you might have looked further.”

### Functionality

A timeline-based structure reflecting the developmental stages of premature infants was suggested as a potential solution to improve content organization. One participant stated in the comment box: “It would also be helpful if there was a timeline showing what a child should do at each (corrected) age.” The ability to share information easily with family members, particularly regarding hygiene practices for visits, emerged as a key functional improvement.

## Discussion

### Overview

This study aimed to develop and evaluate a digital information source (e-TOP) to address the specific information needs of parents caring for preterm infants during the first year postdischarge.

### Development of the App

To develop the e-TOP app, a user-centered design approach was used. The feedback from the 3 focus groups with TOP interventionists and parents of premature infants, including parents with LHL, directly influenced the app’s content and design. The cocreation and iterative process between experts, the design company, and the research team was highly successful in shaping the app’s functionality and refining its design and content. This collaborative approach ensured that the final prototype of the e-TOP app was both user-friendly and rich in relevant, evidence-based information, tailored to meet the diverse needs of its users. This methodology aligns with established practices in mHealth app development, emphasizing the importance of participatory design including health professionals to meet user needs [21-24].

Parents with LHL were actively engaged during the focus group meetings in the developmental phase of the e-TOP app. They expressed a need for clear, actionable information, preferably content in multiple formats, with an accessible tone and simplified messages. The app therefore incorporated features, such as simplified language (Level 1), multimedia content, and “read-out-loud” functionality to accommodate parents with LHL, with videos and text at different reading levels. This preference is supported by research indicating that visual-based interventions, particularly videos, are effective in improving health literacy and comprehension of health-related information [38].

### Usage and Usability

Participants engaged with the app for a median total of 39 minutes (IQR 8.8-53.0) during the 6-month study period. The number of recorded actions per participant had a median of 64.0 (IQR 33.5-88.0), but usage varied significantly between participants. Differences in purpose, target groups, and the lack of user engagement metrics make comparison to other parenting apps challenging, as also described in Li’s systematic review of commercial mHealth parenting apps [39]. The e-TOP app is designed as an additional information source; if we compare the results to educational health apps, longer but less frequent sessions are found [40]. This found gap calls for established standardized benchmarks for digital engagement in neonatal mHealth interventions.

While the app was perceived as useful for finding information on prematurity-specific topics, user data revealed a decline in engagement after the initial 2 weeks. These differences in intent to use versus lower nonusage attrition are found in other web-based parenting interventions [15]. The e-TOP engagement peaked at week 11 and week 16. Parents revisited the app at key developmental milestones, such as rolling over, reaching for toys, and the transition to solid foods. Integrating features such as search functionality, interactive (push notifications and chat function), and timeline-based navigation could enhance the e-TOP app’s long-term utility [41,42].

SUS distribution with a mean SUS score of 68 is considered a suitable usability for Digital Health apps [43]. The e-TOP app demonstrated acceptable-to-good usability with a SUS score of 75.0 (IQR 67.5-80.0). By complementing the SUS with a customized satisfaction survey and data collected through thinking-aloud sessions, interviews, and written comments from the app’s comment box, this study followed best practices of combining quantitative ratings with rich qualitative findings for the usability testing [30]. Participants provided actionable feedback across key areas. By integrating these improvements, the e-TOP app can enhance usability, ensuring it remains a valuable long-term resource for parents of preterm infants.

### Limitations and Opportunities for Future Research

The total study period of 2 years and restricted finances necessitated pragmatic approaches to the evaluation of the usability. We used several qualitative and quantitative methods, but we had to make compromises impacting the study methodology, such as the reliance on a single author for qualitative data analysis which may introduce bias. Integrating multiple researchers into the thematic analysis process not only enriches the analysis through diverse perspectives but also enhances the credibility and trustworthiness of the research findings [44].

Although parents with LHL contributed to the development of the app, the study faced challenges in recruiting parents with LHL for the evaluation phase. Their limited representation in the evaluation phase underscores a broader challenge in engaging these parents in research [45]. Barriers such as complex consent processes and digital questionnaires have hindered participation in our study. Allowing parents to first establish a connection with the researchers or interventionists and using postponed consent is expected to promote inclusivity. Also, verbal consent or simplified forms could foster inclusivity. Although improved comprehension and trust may have positive effects on study enrollment, we emphasize the active involvement of the target group in the early stages of research projects when developing the study design, procedures, and the selection of measurements [46].

This study had a high attrition of the fathers; only 32% of the fathers used the app and 25% completed the usability questionnaires. In general, retaining participants in studies using mHealth apps has been described as challenging [47]. The finding of higher attrition among fathers aligns with the results of the study on the NICU2HOME app (Garfield [18]), in which high usability scores were similarly observed among mothers, but lower engagement among fathers was noted [10]. Xie [48] also found in her systematic review of digital interventions for fathers of infants from conception to 12 months postpartum that fathers have expressed the need and desire for access to relevant, accurate, and up-to-date information on infant care and challenges associated with new parenthood and called for interventions that target father-identified priorities [49]. These studies and our findings highlight the importance of gaining a deeper understanding of perceived barriers to engaging fathers.

The usability evaluation confirms that the app was responsive to the needs of parents with a premature infant. Given the evidence supporting user-centered iterative development, we strongly advocate for proceeding with the implementation of the e-TOP app, ensuring it remains a valuable and available resource while integrating continuous user feedback by using real-time analytics, for example, engagement metrics [50,51]. Future research should focus on assessing the effectiveness by evaluating how app usage translates into improved parental confidence and decision-making in caregiving with the goal of reducing dependency on health care professionals and fostering parental autonomy.

### Conclusion

The e-TOP app has demonstrated strong potential to complement information transfer to the face-to-face care after discharge and to bridge the information gap for parents when they leave the hospital. While usability findings are promising, continued development, customization, and integration of the app with health care systems are necessary to maximize its long-term impact. By addressing user engagement, content expansion, accessibility, and health care integration, future versions of e-TOP can become an essential tool for empowering parents of preterm infants and improving neonatal postdischarge care.

## Supplementary material

10.2196/75569 Multimedia Appendix 1 Focus group outline and key questions.

10.2196/75569 Multimedia Appendix 2 Interview guide for evaluating the e-TOP app.

## Abbreviations

EOP: Dutch Expertise Center for Premature Infants
LHL: limited health literacy
mHealth: mobile health
NICU: neonatal intensive care unit
SUS: system usability scale
