# Supplementary material for: An Information App (e-TOP) to Support Parents’ Information Needs During the First Year at Home After Preterm Birth: Development and Usability Study
Source: JMIR Pediatr Parent. 2025 Oct 22;8:e75569. doi: 10.2196/75569 (PMC12543213; doi:10.2196/75569)
Supplement: Multimedia Appendix 1 [file pediatrics-v8-e75569-s001.docx]

Appendix 1: Focus Group Outline and Key Questions

This appendix outlines the structure and key discussion points used during the focus groups. The sessions were moderated using examples and visual aids to facilitate meaningful conversations.

1. Opening Question

What are your first associations when you hear about an online information source (e.g., an app) for parents of premature infants?

2. Content-Related Questions

a. Practical Concerns Shared by Other Parents

- Do you recognize the following concerns raised by other parents?
 - Why does my baby groan so much during sleep?
 - Until what age should I correct for prematurity?
 - What topics have you personally searched for online?

b. Examples from Other Parents

- “My infant has had a cold for a few days—do I need to consult a doctor?”
- “What can I expect when my child starts school?”
- “Is mental health support available for parents?”
- “What kind of support did you need after returning home from the hospital?”
- Some parents mentioned struggling to build confidence in their parenting role or wished they had received more practical guidance.

c. Sources of Information

- Where do you typically find information?
- Other parents reported seeking advice from healthcare professionals, family, friends, online searches, or Facebook groups.

3. App Development Questions

a. Information Structure Preferences

- Do you prefer information organized by topic or by frequently asked questions? (Mockups were used during this discussion.)

b. Feedback on Mockups

- Are the information cards and texts shown in the mockups clear and sufficient for your needs?
- Different styles of mockups were presented (text-only, images, and combinations).

4. Validation of Text Content

Participants were shown or read sample texts and asked to respond to the following:

a. Open Feedback

- What are your impressions of the text you just read?

b. Rating Statements

Participants rated the text using a 4-point Likert scale (1 = strongly disagree, 4 = strongly agree) for the following statements:
- The text feels personal.
- The text is reassuring.
- The text is alarming.
- The text is informative.

c. Tone of Voice

- What tone of voice do you prefer for such content?
 (Examples were given: healthcare professional, grandmother, peer/buddy.)

5. Preferences for Visual Content

a. Use of Visual Aids

- What are your preferences for visual content (e.g., animations, videos, pictorial stories)?
Examples were presented to the participants to facilitate this discussion.
