# Supplementary material for: An Information App (e-TOP) to Support Parents’ Information Needs During the First Year at Home After Preterm Birth: Development and Usability Study
Source: JMIR Pediatr Parent. 2025 Oct 22;8:e75569. doi: 10.2196/75569 (PMC12543213; doi:10.2196/75569)
Supplement: Multimedia Appendix 2 [file pediatrics-v8-e75569-s002.docx]

Appendix 2: Interview Guide for Evaluating the e-TOP App

1. General Use and Engagement

- Did you use the e-TOP app?

- Could you explain the reasons for using or not using the app?

- How would you describe your overall experience with the e-TOP app?

- Did you use the app during specific moments or situations?

2. Specific Information Needs

- What type of information were you looking for in the app?

- Did the app provide the information you were seeking?

- Can you provide an example of a time when you could not find the information you were looking for?

3. Content and Accessibility

- How did you experience the content provided in the app?

- What were your impressions of the way information was presented (e.g., text, videos)?

- Could you elaborate on whether the level of detail and depth of information was sufficient?

- Were you able to navigate through the chapters to find relevant information?

- Did you find the absence of a search function to be a limitation?

4. Alternatives and Additional Needs

- What did you do when the information you sought was not available in the app?

- Did the videos help you to better understand the content?

- Could you relate to the people or situations shown in the videos?

- What suggestions do you have to improve the app to better meet your information needs?
